# Distinct Mitochondrial Pathologies Caused by Mutations of the Proximal Tubular Enzymes EHHADH and GATM

**DOI:** 10.3389/fphys.2021.715485

**Published:** 2021-07-19

**Authors:** Anna-Lena Forst, Markus Reichold, Robert Kleta, Richard Warth

**Affiliations:** ^1^Medical Cell Biology, Institute of Physiology, University of Regensburg, Regensburg, Germany; ^2^Centre for Nephrology, University College London, London, United Kingdom

**Keywords:** protein aggregates, autosomal dominant mutation, peroxisome, inflammasome, renal fibrosis, mitochondrial damage associated molecular patterns

## Abstract

The mitochondria of the proximal tubule are essential for providing energy in this nephron segment, whose ATP generation is almost exclusively oxygen dependent. In addition, mitochondria are involved in a variety of metabolic processes and complex signaling networks. Proximal tubular mitochondrial dysfunction can therefore affect renal function in very different ways. Two autosomal dominantly inherited forms of renal Fanconi syndrome illustrate how multifaceted mitochondrial pathology can be: Mutation of EHHADH, an enzyme in fatty acid metabolism, results in decreased ATP synthesis and a consecutive transport defect. In contrast, mutations of GATM, an enzyme in the creatine biosynthetic pathway, leave ATP synthesis unaffected but do lead to mitochondrial protein aggregates, inflammasome activation, and renal fibrosis with progressive renal failure. In this review article, the distinct pathophysiological mechanisms of these two diseases are presented, which are examples of the spectrum of proximal tubular mitochondrial diseases.

## Introduction

### Proximal Tubular Tasks and Energy Supply

The renal proximal tubule performs heavy-duty work: it reabsorbs most of the filtered water and salts, and almost all of the glucose, amino acids, and proteins. It is important for calcium, phosphate, and bicarbonate reabsorption and for the pH balance. Moreover, it can secrete metabolic end-products, toxins, and pharmaceuticals into the urine. The proximal tubule is among the segments with the highest content of Na^+^/K^+^ ATPases and is particularly rich in mitochondria. The proximal tubular epithelium has a high paracellular permeability that allows water and substrate fluxes and, thereby, keeps the transepithelial potential small (Frömter and Hegel, 1966; Frömter and Gessner, 1974). Based on micropuncture studies, 1/3 of Na^+^ reabsorption is thought to be directly active (transcellular and energy-dependent), 1/3 occurs through the transepithelial potential, and 1/3 of the reabsorbed Na^+^ is entrained (“solvent drag”) by the paracellular fluid flow (Frömter et al., 1973). Since only a third of Na^+^ leaves the cell directly *via* the Na^+^/K^+^ ATPase in a ATP-consuming manner, proximal tubular Na^+^ reabsorption is very economical: approximately nine Na^+^ ions per ATP can be transported. In comparison, in the collecting duct, all reabsorbed Na^+^ is handled by the Na^+^/K^+^ ATPase, leading to transport of only three Na^+^ ions per ATP (Mandel and Balaban, 1981).

In the kidney, oxygen consumption correlates with Na^+^ reabsorption and only about 15% of the oxidatively produced energy is used for basal metabolism and tubule cell structure maintenance (Deetjen and Kramer, 1961; Deetjen, 1980). Although proximal tubules reabsorb large amounts of glucose, they do not use glucose as a substrate for energy production. They obtain energy almost exclusively by oxidative phosphorylation and use fatty acids, ketone bodies, lactate, and glutamine as energy sources (Mandel, 1985). Thus, the proximal tubule is directly dependent on the supply of oxygen. It is therefore particularly vulnerable in the event of an undersupply of oxygen and nutrients. Mitochondria therefore play a key role in the underlying pathophysiology of ischemic and metabolic kidney injury (Chevalier, 2016).

### Fanconi’s Syndrome

The late part of the proximal tubule (S3) is particularly vulnerable because energy cannot be obtained anaerobically due to the lack of enzymes. The partial pressure of oxygen is also lower in the outer medulla than in the cortex. The proximal tubule is susceptible to damage by toxins and pharmaceuticals, e.g., cadmium, or aminoglycosides, because it takes up and, in some cases, accumulates toxins luminally and/or basolaterally *via* distinctive transporters. Damage of proximal tubular cells inevitably impairs transport processes. According to the first descriptors, the clinical picture is called Fanconi-De-Toni-Debré syndrome (or “renal Fanconi’s syndrome” for short; Fanconi, 1931). This syndrome is characterized by glucosuria, aminoaciduria, phosphaturia, acidosis, and low molecular weight proteinuria. In addition to acquired damage, there are a number of hereditary diseases that directly or indirectly affect the proximal tubule (Bokenkamp and Ludwig, 2011; Klootwijk et al., 2015; Lemaire, 2021). The pathophysiology of these genetic diseases is multifaceted, ranging from defective transport proteins, enzymes, and transcription factors to impaired lysosome function and mitochondrial damage. This review article focuses on two autosomal dominant forms of renal Fanconi’s syndrome, both of which affect mitochondria. However, the underlying pathomechanisms are quite different and exemplify the spectrum of mitochondrial diseases.

### Mitochondria – More Than Just Power Plants

According to the endosymbiont hypothesis, mitochondria are organelles that arose from the uptake of an α-proteobacterium by a (pre-)eukaryotic cell (Gray, 2015). Mitochondria have an outer and an inner membrane separating the intermembrane space from the matrix. The outer membrane is highly permeable, whereas the inner membrane with its infoldings (cristae) represents a barrier that requires specific transport processes to overcome. Oxidative phosphorylation in the respiratory chain generates the proton gradient across the inner membrane, which ultimately drives ATP synthesis. Mitochondria are highly dynamic organelles; complex processes of fusion and fission regulate the mitochondrial network, cristae remodeling and, eventually, cytochrome-*c* release that can result in apoptosis (Scorrano et al., 2002; Westermann, 2010). Mitochondria have a circular genome (mtDNA), which encodes 13 proteins involved in oxidative phosphorylation and genes for ribosomal RNAs and transfer RNAs. The remainder of the approximately 1,140 mitochondrial proteins are encoded by genes of the cell nucleus.^1^ These proteins are imported into mitochondria after or during protein synthesis at cytosolic ribosomes. Of note, mitochondria have cell-type specific properties: The protein composition of mitochondria and consequently their functional properties – besides ATP synthesis – are highly dependent on the cellular context and adapted to the respective needs.

Mitochondria produce the majority of cellular energy by combining the oxidation of nutrients *via* the respiratory chain with ATP synthesis. In addition to ATP production, mitochondria have essential metabolic roles and contribute to intracellular Ca^2+^ homeostasis, pyrimidine and heme synthesis, amino acid metabolism, beta-oxidation pathway, urea cycle, steroid hormone synthesis, and thermogenesis. Mitochondria are signaling hubs and influence processes, such as programed cell death, inflammation, and innate immunity (West, 2017). Moreover, mitochondria influence basic cellular functions, such as gene transcription, and, on the other hand, are embedded in and adapted to the specific cellular context. When damaged, mitochondria trigger a plethora of signals that can result in an inflammatory response, a transcriptional response, and/or the initiation of specific forms of cell death (Bahat and Gross, 2019). Mitochondria are therefore relevant in acquired or hereditary kidney damage as well as in repair processes.

### Mitochondrial Pathologies

Disruption of normal mitochondrial function, especially ATP production, is the main cause of a group of heterogeneous genetic diseases, the mitochondriopathies. They are clinically diverse, but often result in functional impairment of muscle or nerve cells. In most cases, mutations in genes involved in oxidative phosphorylation or mitochondrial metabolism are the molecular cause of these diseases. Some mitochondriopathies affect the kidneys in addition to impaired function of other organs, and some even affect the proximal tubule of the kidney to a particular extent (Emma et al., 2012, 2016; Srivastava et al., 2020). Some factors, such as mitochondrial sirtuins, act as metabolic sensors and appear to have modulatory effects on cell apoptosis, inflammation, fibrosis, and adaptive responses to stress (Morigi et al., 2018). Due to its strong dependence on oxidative energy production and specific metabolic pathways, the proximal tubule is susceptible for pathological mutations of genes encoding its specific subset of mitochondrial proteins; however, the same mutated proteins might be irrelevant in other kidney segments or other tissues. Table 1 shows examples of mitochondrial diseases affecting the kidney. Moreover, in recent years, large-scale genome-wide association studies have led to the identification of many gene loci associated with renal function (Teumer et al., 2019; Wuttke et al., 2019; Schlosser et al., 2020; Gorski et al., 2021). Some of these loci contain genes encoding proteins that are imported into mitochondria (Table 2).

**Table 1:** Examples of renal mitochondriopathies.

Disease (OMIM number)	Affected gene	Mechanism	Leading symptoms	Expression^1^	References
COQ2 Nephropathy (609825, 607426)	*COQ2*	Impaired function of the para-hydroxybenzoate-polyprenyl-transferase resulting in defective synthesis of coenzyme Q (CoQ10), or ubiquinone, a mobile lipophilic electron carrier critical for electron transfer by the mitochondrial inner membrane respiratory chain.	Clinically heterogeneous autosomal recessive syndrome with encephalopathy, epilepsy, glomerular lesions, steroid-resistant nephrotic syndrome, and progressive renal failure.	S1, S2, thin limbs, IMCD	Rotig et al., 2000; Diomedi-Camassei et al., 2007
Renotubular Fanconi’s syndrome 3; FRTS3 (615605)	*EHHADH*	Mistargeting of peroxisomal EHHADH into mitochondria impairs mitochondrial metabolism and oxidative phosphorylation in the proximal tubule.	Autosomal dominant isolated Fanconi’s syndrome (phosphaturia, glucosuria, aminoaciduria, metabolic acidosis, and low molecular weight proteinuria) without obvious impairment of glomerular function.	S2, S3	Tolaymat et al., 1992; Klootwijk et al., 2014; Assmann et al., 2016
Renotubular Fanconi’s syndrome 1; FRTS1 (134600) with kidney failure	*GATM*	Formation of large intramitochondrial fibrils containing mutated GATM. The fibrils escape degradation and elicit signals that drive inflammation, epithelial cell death, and fibrosis.	Autosomal dominant renal Fanconi’s syndrome early in life (phosphaturia, glucosuria, aminoaciduria, metabolic acidosis, and low molecular weight proteinuria) with impairment of glomerular function later in life.	S1, S2	Lichter-Konecki et al., 2001; Reichold et al., 2018; Wuttke et al., 2019
VATER/VACTERL association (192350) and Congenital abnormalities of the kidney and urinary tract (CAKUT)	*TRAP1*	TRAP1 is a mitochondrial chaperone (heat-shock protein 90-related). It might be involved in antiapoptotic and endoplasmic reticulum stress signaling.	VATER/VACTERL association: Nonrandom association of vertebral defects (V), anal atresia (A), tracheoesophageal fistula with esophageal atresia (TE), and radial or renal dysplasia (R). CAKUT: Congenital abnormalities of the kidney and urinary tract. Gene locus associated with kidney function.	Whole nephron	Saisawat et al., 2014; Kause et al., 2019; Wuttke et al., 2019

S1, first segment of the proximal tubule; S2, mid-segment of the proximal tubule; S3, late and straight segment of the proximal tubule; thin limbs, thin limbs of the loop of Henle; and IMCD, inner medullary collecting duct. Additional examples of mitochondriopathies affecting the kidney can be found in specialized reviews (Finsterer, 2004; O’Toole, 2014; Finsterer and Scorza, 2017).

1 Expression according to: https://esbl.nhlbi.nih.gov/MRECA/Nephron/, https://helixweb.nih.gov/ESBL/Database/NephronRNAseq/All_transcripts.html, https://cello.shinyapps.io/kidneycellexplorer/.

**Table 2:** Examples of gene loci of mitochondrial proteins associated with kidney function.

Gene or locus	Expression^1^	Leading symptom or pathophysiology	References
MRPS30	S1, S2	Gene locus associated with kidney function.	Wuttke et al., 2019
SLC25A29	All nephron segments except S1 and S2	Gene locus associated with kidney function.	Wuttke et al., 2019
CPS1	All nephron segments except S1 and S2	Gene locus associated with kidney function.	Wuttke et al., 2019
RDH14	Ubiquitous, S1, S2	Gene locus associated with kidney function.	Wuttke et al., 2019
DMGDH	S2, S3	Gene locus associated with kidney function. Potentially linked to renal ischemia-reperfusion injury.	Zhu et al., 2018; Wuttke et al., 2019
MSRA	S1, S2	Gene locus associated with kidney function. Possible role during ischemia-reperfusion injury and renal fibrosis.	Kim et al., 2013, 2015; Wuttke et al., 2019
AGMAT	S1, S2	Gene locus associated with kidney function. Possible biomarker for diabetic glomerulopathy and possible role in renal cancer.	Dallmann et al., 2004; Hsu et al., 2015; Wuttke et al., 2019
CYP24A1	S1	Gene locus associated with kidney function. Vitamin D metabolism.	Chesney, 2016; Wuttke et al., 2019
SND1	S1, S2	Gene locus associated with kidney function. Possible role in renal clear cell carcinoma.	Wuttke et al., 2019; He et al., 2020
MYO19	All nephron segments except S1 and S2	Gene locus associated with kidney function.	Wuttke et al., 2019
CEP89	S1, S2	Gene locus associated with kidney function.	Wuttke et al., 2019

S1, first and convoluted segment of the proximal tubule; S2, mid-segment of the proximal tubule; and S3, late and straight segment of the proximal tubule.

1 Expression according to: https://esbl.nhlbi.nih.gov/MRECA/Nephron/, https://helixweb.nih.gov/ESBL/Database/NephronRNAseq/All_transcripts.html, https://cello.shinyapps.io/kidneycellexplorer/.

## A Hereditary Fanconi’s Syndrome Caused by Reduced Energy Supply

In hereditary forms of renal Fanconi’s syndrome, gene mutations disrupt the structure and function of the affected proteins and consequently impair the function of proximal tubular cells. Depending on the extent of the functional impairment and the relevance of the affected gene, the impairment can range from mild and circumscribed dysfunction to severe cell damage, cell death, and consecutive fibrosis with progressive loss of renal function. The molecular changes induced by the mutation may lead to misfolding, premature protein degradation, loss of protein function, or the misfolded protein may develop a pathological function, interfere with other cellular processes, or escape degradation by aggregate formation, thereby damaging cells.

### EHHADH: Impaired Oxidative Phosphorylation Caused by a Mistargeted Protein

Our studies in an extended family with autosomal dominant renal Fanconi’s syndrome and severe rickets provided an opportunity to investigate the underlying pathophysiological mechanisms (Klootwijk et al., 2014). Affected family members had isolated renal Fanconi’s syndrome in which reabsorption of phosphate, glucose, bicarbonate, amino acids, and low molecular weight proteins was impaired. Impaired function of other organs was not found except for impaired growth and bone demineralization due to vitamin D-resistant rickets, which is a consequence of renal phosphate loss and metabolic acidosis. Genetic analysis showed that affected patients, but not healthy family members, had a heterozygous pathogenic mutation (p.E3K) in *EHHADH*. This gene encodes the bifunctional peroxisomal enzyme “enoyl-CoA hydratase-l-3-hydroxyacyl-CoA dehydrogenase,” also known as l-bifunctional enzyme or l-PBE. This enzyme is involved in peroxisomal beta-oxidation of straight-chain saturated acyl-CoA fatty acids.

In the fatty acid beta-oxidation pathway, the first reaction is accomplished by fatty acid acyl-CoA oxidase, the second and third reactions are accomplished by EHHADH, and the last step is catalyzed by 3-ketoacyl-CoA thiolase. Another bifunctional enzyme, d-bifunctional enzyme (d-PBE, HSD17B4), can accomplish the second and third steps of the beta-oxidation pathway similar to EHHADH. The role of EHHADH for fatty acid and energy metabolism is not an essential one. This is supported by the fact that *Ehhadh* knockout mice are viable and display no obvious phenotype (Houten et al., 2012). In our studies, we took a closer look at proximal tubular function in *Ehhadh* knockout mice but found neither evidence for disturbance of proximal tubular function nor impaired renal energy metabolism. This indicated that the disruption of the physiological function of EHHADH in peroxisomes is not disturbing normal kidney function in *Ehhadh* knockout mice.

These data also argued against haploinsufficiency and reduced peroxisomal fatty acid oxidation as the cause of the disease in our patients. Rather, these data suggested that the mutant protein interferes with proximal tubule function in other ways. *In silico* analysis^2^ of the mutant protein suggested a surprising mechanism: The N-terminal mutation p.E3K in our patients gives rise to a pathological mitochondrial targeting signal, and the C-terminal targeting motif for the peroxisome was conserved. Studies on cells expressing the mutant EHHADH revealed that indeed the mutant EHHADH was no longer found exclusively in peroxisomes but mainly in mitochondria. Mistargeting from peroxisomes to mitochondria as a cause had already been described in the literature in another disease, autosomal recessive primary hyperoxaluria (Danpure, 2006). However, in the latter disease, the absence of the mutant enzyme in the peroxisome is crucial for disease development. The fact that *Ehhadh* knockout mice do not have a renal phenotype suggested that it is not the absence of Ehhadh in the peroxisome that is crucial. Measurements of oxidative phosphorylation by oximetry in cells over-expressing mutant EHHADH showed that indeed mitochondrial function and ATP synthesis were decreased. The hypothesis of mitochondrial functional impairment as a disease-causing mechanism is supported by the increased excretion of mitochondrial metabolites in the urine of our patients.

**Figure 1:**
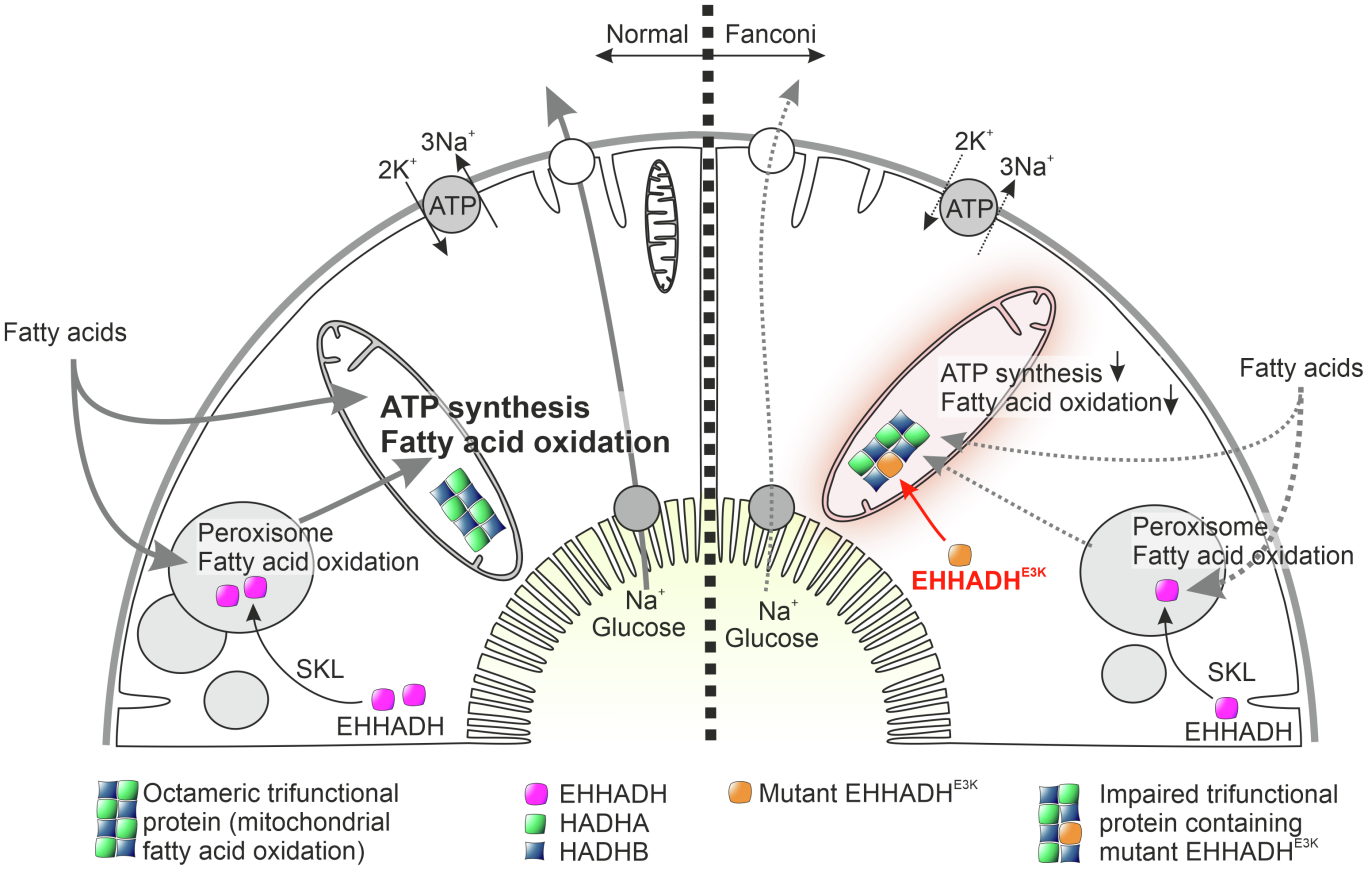
Working hypothesis on the pathophysiology of mutant EHHADH-induced Fanconi’s syndrome without kidney failure. The physiological function of normal EHHADH is shown on the left, and the situation in cells carrying the EHHADH^E3K^ mutation is shown on the right. Normally, EHHADH is imported into peroxisomes based on a C-terminal targeting sequence (SKL motif), where it plays a role in beta-oxidation of long fatty acids. The N-terminal missense mutation of EHHADH found in our patients generates a mitochondrial targeting signal that leads to pathological import of mutant EHHADH into mitochondria. In the mitochondrial matrix, mutant EHHADH presumably replaces HADHA within the octameric trifunctional protein, an enzyme complex required for mitochondrial fatty acid oxidation. Like a faulty component in a machine, mutant EHHADH thus interferes with mitochondrial fatty acid degradation and ATP synthesis. Proximal tubular cells, which normally transport large amounts of substrates and water under ATP consumption, are therefore no longer able to maintain their normal reabsorption capacity. As a result, glucose, amino acids, bicarbonate, and low molecular weight proteins are lost to the urine. Interestingly, proximal tubular cells do not appear to be further damaged by impaired mitochondrial fatty acid degradation and no damage-associated proinflammatory signals are generated.

Further investigation of the pathomechanism revealed that mutant EHHADH is incorrectly incorporated into the so-called trifunctional protein (MTP), which is responsible for mitochondrial beta-oxidation of long-chain fatty acids. Like a false cog in a gearbox, the mutant EHHADH thereby impairs the function of MTP and an accumulation of hydroxyacyl- and acylcarnitines occurs (Assmann et al., 2016). In addition, evidence was found for impaired formation of supercomplexes of respiratory chain enzymes. The perturbations of mitochondrial function collectively resulted in decreased oxidative phosphorylation capacity, impaired mitochondrial membrane potential, and, finally, decreased ATP generation, which reduced tubular transport. Interestingly, however, no evidence was found for the initiation of fatal mitochondrial signals that could have led to inflammation or cell death. Consistent with this, affected patients show a clear reabsorption deficit of the proximal tubule but no progressive loss of renal function (Figure 1).

Why, however, is there no disturbance of the function of the liver, which also strongly expresses EHHADH? The explanation may lie in the unique energy production of proximal tubular cells and their particular dependence on mitochondrial fatty acid oxidation. The decrease of ATP synthesis in this context impairs transport function, but ATP levels are still sufficient for structural maintenance of the proximal tubule. Hepatocytes, on the other hand, do not appear to be as dependent on mitochondrial fatty acid oxidation despite their high expression of EHHADH, and liver function defects were indeed not described in patients.

## Consequences of Mitochondrial Protein Aggregates

### Disease-Causing Aggregates

Human diseases attributable to protein aggregate formation include Alzheimer’s disease (Eftekharzadeh et al., 2018; Kollmer et al., 2019) and Lewy body dementia (Outeiro et al., 2019), with cellular damage related to cytoplasmic accumulation of toxic proteins (Selkoe, 2003). Pathological protein and non-protein aggregates can also lead to profound cellular damage in the kidney, particularly in the proximal tubule, if the cells are unable to degrade or eliminate these aggregates. For example, in cystinosis, crystal-like cystine aggregates are found in proximal tubular cells, providing the starting point for tubular damage and driving the disease process (Cherqui and Courtoy, 2017). Crystal-like protein aggregates have also been described as a possible pathomechanism of the renal disease in multiple myeloma. In these disorders, it is monoclonal immunoglobulin light chains that are responsible for aggregate formation, leading to tubular injury, interstitial inflammation, fibrosis, and tubular atrophy (Sirac et al., 2021). Another example of the severe tubular damage that can be induced by crystals is oxalate-induced acute kidney injury. The pathogenesis and signaling pathways in oxalate-induced necroinflammation are complex: Recent data suggest that activation of inflammation, necroptosis, and “mitochondrial permeability transition-regulated necrosis” play a role (Mulay et al., 2019). Recently, we have described a previously unknown kidney disease in which unique aggregates of a mutant GATM protein form directly in mitochondria. These aggregates lead to giant, elongated mitochondria and eventually trigger signals that lead to tubular damage, renal Fanconi syndrome, and later in life to fibrosis and progressive loss of renal function (Reichold et al., 2018).

### GATM: Mitochondrial Mutant Protein Aggregates Cause Cell Damage, Inflammation, and Fibrosis

During childhood, our patients showed renal Fanconi’s syndrome with glucosuria, phosphaturia, aminoaciduria, low molecular weight proteinuria, and metabolic acidosis, but no significant rickets or bone deformities. In late adolescence or adulthood, they developed renal fibrosis and renal function loss leading to dialysis requirement in the third to sixth decade of life (Reichold et al., 2018).

Genetic analysis revealed that all patients had heterozygous missense mutations in one gene, the “glycine amidinotransferase” gene, GATM (also called “l-arginine:glycine amidinotransferase,” AGAT). In each family, one variant segregated with the disease and was fully penetrant. None of the unaffected family members carried any of these GATM mutations. As a mitochondrial protein, GATM transfers a guanidino group from l-arginine to glycine, resulting in guanidinoacetate, the immediate precursor of creatine. Gene expression of GATM is highest in liver and kidney,^3^^,^^4^ especially in the early segments of the proximal tubule.^5^

How do our patients’ GATM mutations cause kidney disease? Homozygous or compound heterozygous loss-of-function mutations of GATM cause “cerebral creatine deficiency syndrome,” a rare congenital defect in creatine synthesis characterized by severe neurological impairment but not obvious renal dysfunction (Clark and Cecil, 2015). Also, a *Gatm* knockout mouse model shows neurological symptoms due to creatine deficiency but normal renal function and no evidence of renal fibrosis (Choe et al., 2013). In contrast, our patients showed no extra-renal symptoms and no creatine deficiency. Clues to the pathogenesis of the diseases were obtained by examining renal biopsies from affected individuals: First, the biopsies showed renal fibrosis; second, proximal tubules showed drastically enlarged mitochondria containing fibril-like structures. Immunogold staining to label GATM showed that these fibrils contained GATM. In a kidney specimen from a GATM patient with end-stage renal disease, proximal tubules were almost completely gone and replaced by scar tissue indicating that the damage of proximal tubules finally results in the death of cells and nephrons (Reichold et al., 2018). Next, we transfected LLC-PK1 cells (a porcine proximal tubular cell line) with the various mutants of GATM detected in the patients. All of these mutants were found to have enlarged and elongated GATM-positive mitochondria, albeit to varying degrees. The molecular cause of fibril formation by mutant GATM appeared to lie in the nature and localization of the mutations: The mutations created new “interaction sites” that allowed the dimeric GATM protein to multimerize and form fibrils. The occurrence of protein aggregates has already been described in the so-called “mitochondrial myopathies” (Farrants et al., 1988; O’Gorman et al., 1997), but has not yet been observed in kidney cells.

Interestingly, the half-life of the mutant protein present in fibrils was considerably increased, suggesting impaired degradation of fibrillary protein and whole mitochondria by mitophagy and presumably may lead to over-aging of mitochondria (Reichold et al., 2018). Moreover, oxidative phosphorylation in the cells with mutant GATM was largely identical to that in cells expressing the wild-type protein.

**Figure 2:**
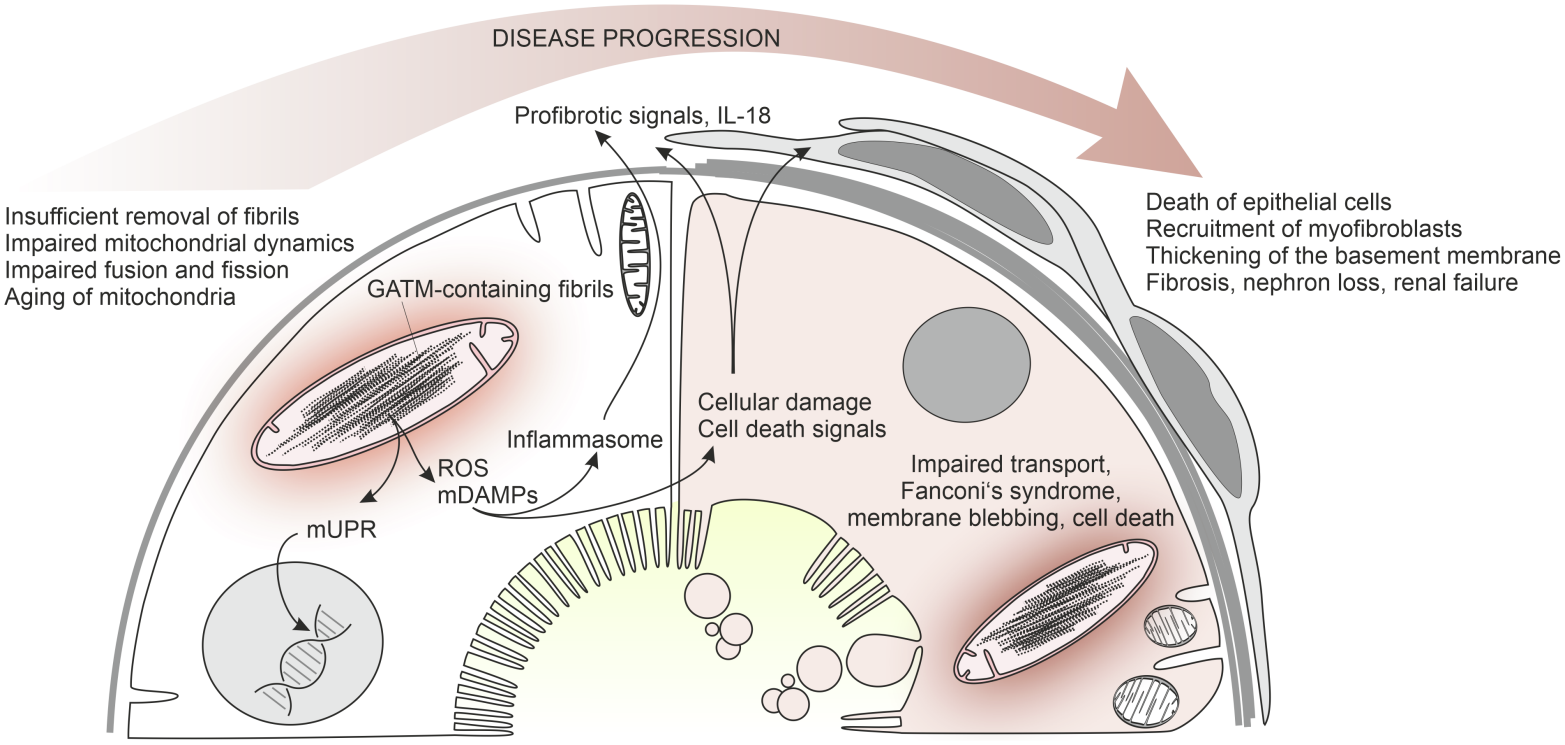
Working hypothesis of the pathophysiology of mutant GATM-induced Fanconi’s syndrome with kidney failure. An early state of the disease is shown on the left, a later state on the right. Mutated GATM forms fibrillary structures within the mitochondrial matrix that cannot be degraded. In LLC-PK1 cells, these large pathological mitochondria are associated with increased ROS production, increased NLRP3 expression, strongly increased IL-18 production, and increased mRNA for fibronectin and alpha smooth muscle actin (Reichold et al., 2018). Presumably, release of mitochondrial danger-associated molecular patterns (mDAMPs) underlies the activation of the inflammasome. Moreover, mitochondrial unfolded protein response possibly occurs leading to transcriptional changes. The persistent mitochondrial fibrils impair mitochondrial dynamics, fusion, and fission of mitochondria and result in mitochondrial aging and enhanced cell death (right side of the model). Profibrotic mediators, such as IL-18, and factors released from dying cells activate interstitial cells and myofibroblasts are recruited. In our patients with end-stage renal disease, proximal tubules are almost absent and have been replaced by scar tissue.

The question therefore arose why tubule cells are damaged in such a way that, in addition to disturbed transport processes, signals are generated that result in inflammation and fibrosis formation. Indeed, we observed that cells with such pathological mitochondria produce more reactive oxygen species. Furthermore, transcription of NLRP3 as an inflammasome component was increased, as well as that of fibronectin and smooth muscle actin, and IL-18 was produced as a proinflammatory factor (Reichold et al., 2018). These findings strongly suggest that mitochondrial GATM aggregates lead to activation of inflammasome components and release of profibrotic factors, thus establishing a plausible pathogenic link between heterozygous GATM mutations, renal fibrosis, and renal failure (Figure 2).

Because mutant GATM protein leads to pathogenic intramitochondrial deposition in renal proximal tubule cells, we investigated means to reduce GATM production. Because GATM expression in rats is negatively regulated by creatine (McGuire et al., 1984), we supplemented wild-type mice with 1% creatine in drinking water for 1 week. This protocol reduced renal GATM mRNA expression by 27% and GATM protein by 58% (Reichold et al., 2018). Therefore, creatine supplementation could serve as an intervention to suppress endogenous production of mutant GATM protein and delay the formation of deleterious mitochondrial deposits. However, further studies will be necessary to validate this therapeutic strategy in preclinical and clinical settings.

## Discussion

The causes of renal Fanconi’s syndrome are multifaceted, and often, it is a sign of severe damage of proximal tubules. Here, we have summarized the pathophysiology of two forms of autosomal dominant Fanconi’s syndrome. In patients carrying mutant EHHADH, the disease is caused by mistargeting of the mutant protein into mitochondria. Usually localized in peroxisomes and involved in fatty acid beta-oxidation, the EHHADH mutation generates an N-terminal mitochondrial targeting signal. When mistargeted into mitochondria, EHHADH associates with and impairs the fatty acid oxidation machinery of mitochondria, and reduces mitochondrial membrane potential and ATP production leading to diminished proximal tubular reabsorptive function (Klootwijk et al., 2014, 2015; Assmann et al., 2016). In these patients, the Fanconi-type transport deficit is not paralleled by progressive loss of kidney function indicating that the reduction of mitochondrial capacity for ATP production alone is not necessarily associated with mitochondrial signals leading to an inflammatory response, cell death, and fibrosis. Probably, impairment of mitochondrial fatty acid oxidations puts proximal tubular cells “on a diet” and ATP synthesis is reduced without causing substantial mitochondrial stress.

The pathophysiology of the GATM-associated mitochondrial disease expands the spectrum of diseases associated with pathological protein aggregates. Compared with the EHHADH-linked Fanconi’s syndrome, the clinical situation of patients carrying those GATM mutations is quite the opposite: The symptoms typically found in Fanconi’s syndrome are rather mild, e.g., vitamin d-resistant rickets is absent or very mild. However, the GATM-linked disorder is characterized by progressive loss of glomerular filtration rate later in life; dialysis is required in the third to sixth decade of life (Reichold et al., 2018). Thus, this disease highlights the critical role mitochondria can play in initiating devastating profibrotic signaling cascades – even in a situation where ATP production appears being unaffected.

Further investigation of the differences between the two disease mechanisms may therefore provide insights into the conditions under which mitochondrial signaling can have particularly severe consequences (Hill et al., 2018; Tremblay and Haynes, 2020). An interesting observation is the extremely prolonged half-life of the fibrillar mutant GATM protein which is – in part – reminiscent of the role of mitochondria in aging (Terman and Brunk, 2006; Shpilka and Haynes, 2018). Or to say it with a phrase taken from an excellent review (Ashrafi and Schwarz, 2013): “The mitochondrion is an important factor in the life of a eukaryotic cell, but, like all good actors, a mitochondrion needs to exit the stage at the right time.” Normal mitochondrial dynamics with fusion and fission events is considered important for the maintenance of mitochondrial functions (Westermann, 2010). Due to the mitochondrial dynamics, it is difficult to estimate turnover rates of mitochondria, but most likely, it is in the range of 2–4 weeks (Terman and Brunk, 2006; Terman et al., 2010). The transfected wild-type GATM protein is largely degraded by LLC-PK1 cells within 2 weeks. In contrast, the mutant protein is still detectable in the cells in the form of large fibrils even after 10 weeks. What are possible explanations for this phenomenon? Several pathways exist for the degradation of misfolded mitochondrial proteins, which normally leads to the disappearance of defective proteins: First, mitochondria are able to degrade proteins by protease complexes. A second pathway involves vesicle-based transfer of damaged proteins to cellular lysosomes. The so-called mitophagy is the third possibility. In mitophagy, an entire mitochondrion is enclosed by an autophagosome, sequestered, and subsequently fused to a lysosome and degraded (Ashrafi and Schwarz, 2013; Dombi et al., 2018; Yoo and Jung, 2018). Obviously, these mechanisms are overwhelmed with the degradation of the fibrils and apparently even mitophagy does not seem to be sufficiently able to degrade the pathologically enlarged mitochondria (Terman et al., 2010). By contrast, in the above-mentioned EHHADH-linked disease, although the mutant protein is mistargeted to mitochondria, there is no progressive accumulation of the mutant protein over time.

Taken together, mutant GATM-containing fibrils appear to elicit a plethora of dangerous signals and events: Generation of reactive oxygen species, the so-called “mitochondrial unfolded protein response,” release of “mitochondrial damage-associated molecular patterns” (mDAMPs), transcriptional changes, inflammasome activation, profibrotic signals, severe cellular damage, and cell death (Terman and Brunk, 2006; Terman et al., 2010; Zorov et al., 2014; Grazioli and Pugin, 2018; Hill et al., 2018; Reichold et al., 2018; Shpilka and Haynes, 2018; Lin et al., 2019). Also noteworthy in this context is the fact that transfected LLC-PK1 cells begin to express fibronectin and smooth muscle actin in addition to inflammasome activation and production of the profibrotic IL-18. Histology of kidney sections of a patient showed proximal tubules with thickened basement membrane and surrounded by connective tissue cells. We therefore assume that the activation of interstitial cells by signals from tubular epithelial cells is the main driver for fibrosis rather than the complete conversion of epithelial cells into connective tissue cells.

EHHADH- and GATM-related hereditary kidney diseases are rare disorders. Thus, the question arises as to how relevant these disorders are to understanding chronic kidney disease. On the one hand, elucidating the underlying causes of disease allows insights into signaling pathways that contribute to the development of kidney disease in general. For example, the mechanisms driving renal fibrosis are of particular interest in this context, as they, together with necroinflammation, nephron loss, and tubular atrophy, represent the common end pathway of a variety of diseases (Bohle et al., 1987; Mulay et al., 2016). On the other hand, forms of “autosomal dominant tubulointerstitial kidney disease” are likely underdiagnosed to a considerable extent, as too often patients are not examined by physicians until the end stage of kidney disease, at which point genetic diagnosis is no longer sought due to irreversibly fibrotic kidneys (Eckardt et al., 2015; Knaup et al., 2018).

Why are diseases involving mitochondria often particularly complex and variable in terms of the tissues affected and the severity of the pathology? As mentioned earlier, the mitochondrial protein composition and function vary widely and are adapted to the requirements of each cell. Moreover, the energy production in the different segments and cell types of the kidney are divers and influenced by the large oxygen gradient between cortex, medulla, and papilla. The segment-specific properties of mitochondria underly functional adaptation, but they also lead to differences in susceptibility to genetic and non-genetic causes of mitochondrial dysfunction. In addition, genes encoded by the mitochondrial genome exhibit peculiarities: mtDNA inherited from the mother encodes essential genes of energy metabolism and is present in thousands of copies per cell. Compared to nuclear DNA, mitochondrial DNA has a high mutation rate. Mutated mtDNA is therefore mixed with “normal” mtDNA within a given cell, a condition known as “heteroplasmy” (Wallace and Chalkia, 2013). The functional consequences of heteroplasmy depend on the degree of accumulation of the mutated mtDNA, the nature of the mutation, and the particular sensitivity of the affected cells for the mutational consequences. However, renal diseases caused by mutations of mitochondrial DNA were not the focus of this review and interested readers are referred to a recent review (Govers et al., 2021).

It is also important not to consider mitochondria as isolated organelles: The crosstalk between mitochondria, cytosol, endoplasmic reticulum, and nucleus is of significant importance for understanding acute and chronic kidney diseases (Mulay et al., 2016; Romagnani et al., 2017; Hill et al., 2018). This hypothesis is underlined by the pathophysiology of mitochondriopathies affecting the kidney (O’Toole, 2014; Finsterer and Scorza, 2017; Lemaire, 2021; examples given in Table 1) and by gene loci of nuclear-encoded mitochondrial proteins^6^ that are associated with kidney function in genome-wide association studies (Wuttke et al., 2019; Gorski et al., 2021; examples given in Table 2).

Over the last years the complexity of mitochondrial functions and their particular importance in the development of renal diseases have become clearer. Rapidly developing technological capabilities for genetic testing, analysis of genetic and non-genomic disease mechanisms and new biomarkers will help to make the correct diagnosis in a larger number of patients and at an earlier disease state (Schlosser et al., 2020; Erdbrugger et al., 2021; Hay et al., 2021; Tin and Kottgen, 2021). Challenging tasks for the future include deciphering the complex mitochondrial signals and signaling pathways that determine adaptation and regeneration on the one hand and maladaptation, cell death, fibrosis, and renal failure on the other hand.

## Author Contributions

All authors listed have made a substantial, direct and intellectual contribution to the work, and approved it for publication.

### Conflict of Interest

The authors declare that the research was conducted in the absence of any commercial or financial relationships that could be construed as a potential conflict of interest.

## Footnotes

^1^https://www.broadinstitute.org/mitocarta/mitocarta30-inventory-mammalian-mitochondrial-proteins-and-pathways

^2^http://bioinf.scmb.uq.edu.au:8080/pprowler_webapp_1-2/

^3^https://www.ncbi.nlm.nih.gov/gene/2628

^4^https://gtexportal.org/home/gene/GATM

^5^https://esbl.nhlbi.nih.gov/MRECA/Nephron/

^6^https://www.broadinstitute.org/mitocarta/mitocarta30-inventory-mammalian-mitochondrial-proteins-and-pathways
